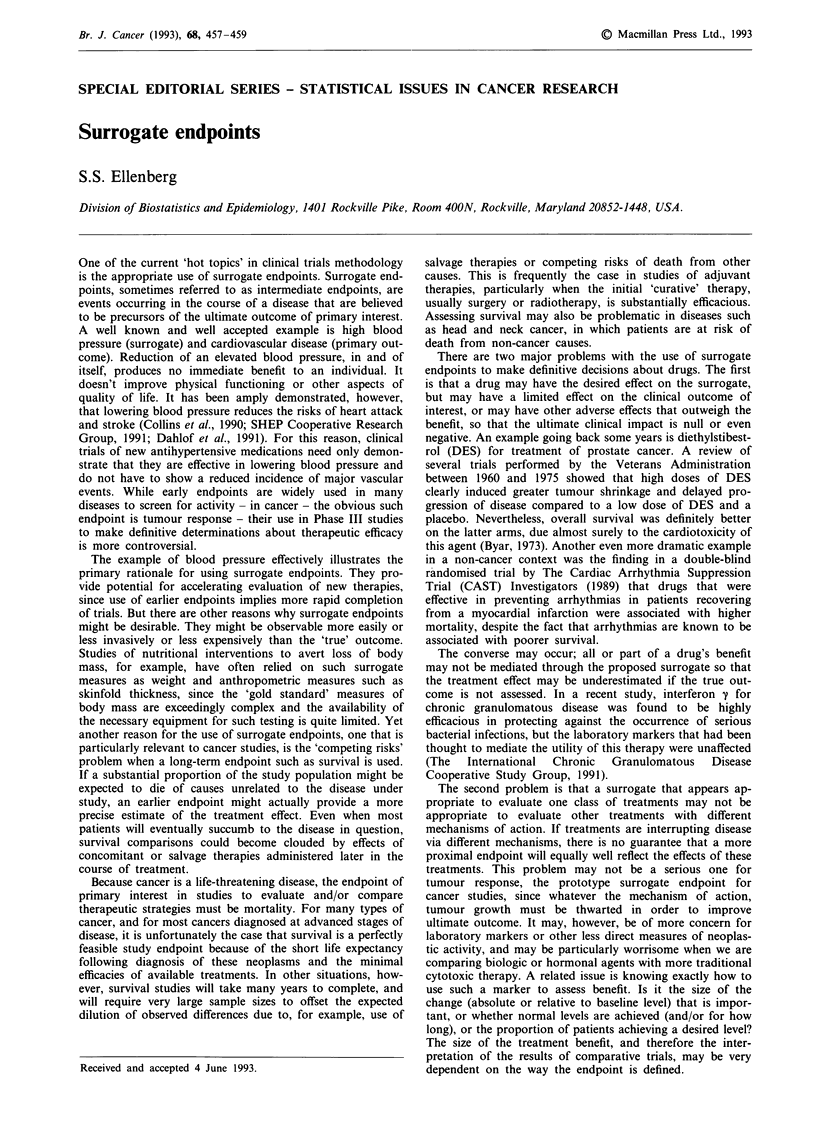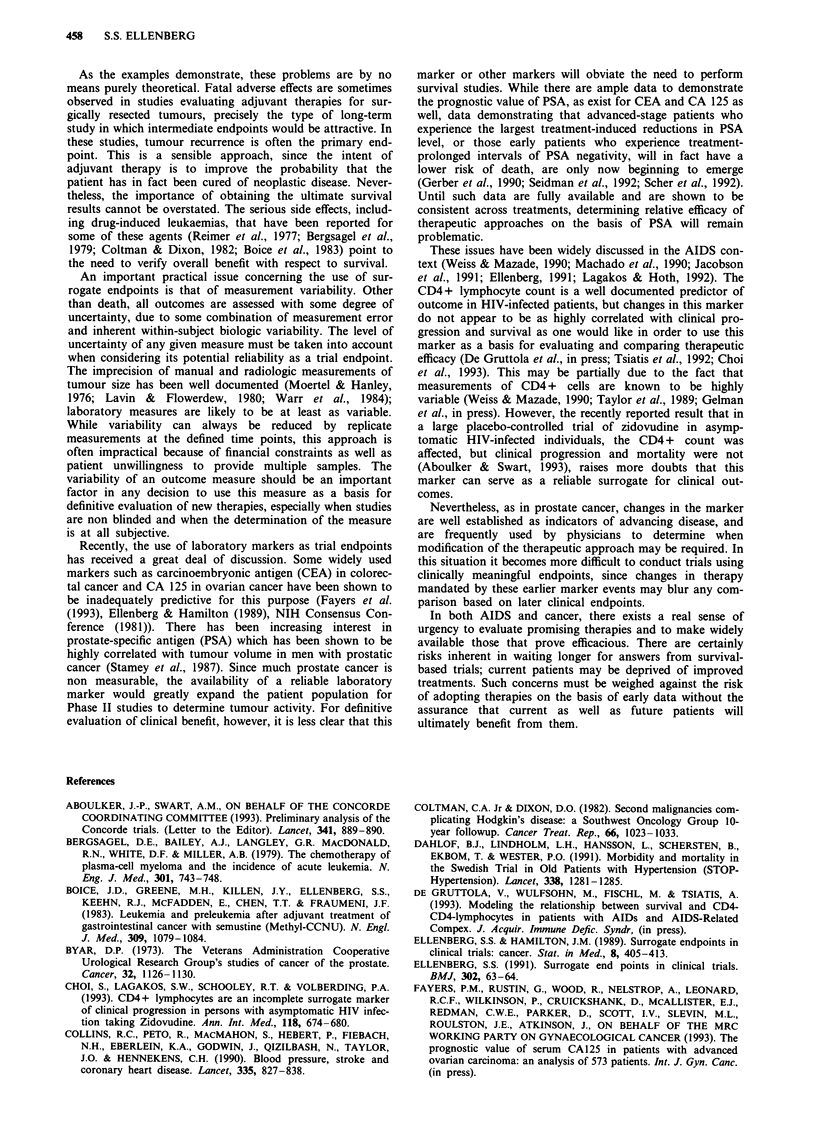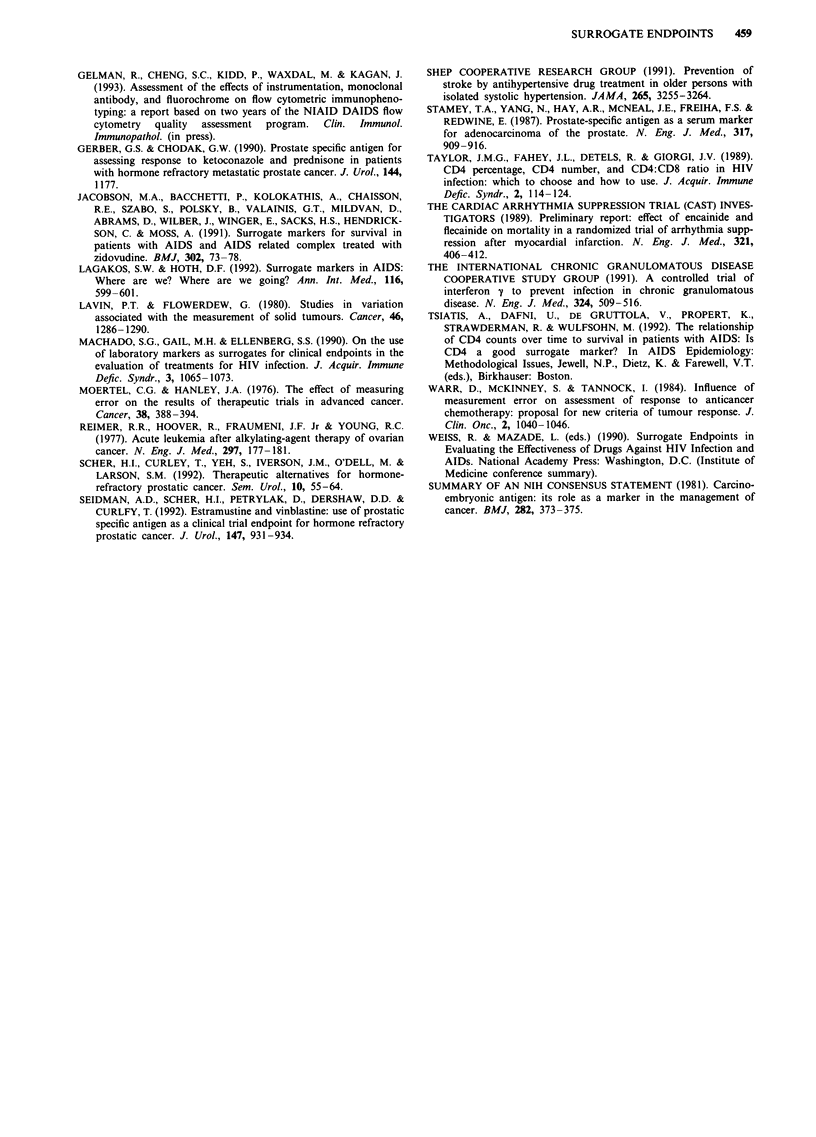# Surrogate endpoints.

**DOI:** 10.1038/bjc.1993.369

**Published:** 1993-09

**Authors:** S. S. Ellenberg


Br. J. Cancer (1993), 68, 457–459 © Macmillan Press Ltd., 1993

SPECIAL EDITORIAL SERIES - STATISTICAL ISSUES IN CANCER RESEARCH

### Surrogate endpoints

S.S. Ellenberg

*Division of Biostatistics and Epidemiology, 1401 Rockville Pike, Room 400N, Rockville, Maryland 20852-1448, USA.*

One of the current 'hot topics' in clinical trials methodology is the appropriate use of surrogate endpoints. Surrogate endpoints, sometimes referred to as intermediate endpoints, are events occurring in the course of a disease that are believed to be precursors of the ultimate outcome of primary interest. A well known and well accepted example is high blood pressure (surrogate) and cardiovascular disease (primary outcome). Reduction of an elevated blood pressure, in and of itself, produces no immediate benefit to an individual. It doesn't improve physical functioning or other aspects of quality of life. It has been amply demonstrated, however, that lowering blood pressure reduces the risks of heart attack and stroke (Collins *et al.*, 1990; SHEP Cooperative Research Group, 1991; Dahlof *et al.*, 1991). For this reason, clinical trials of new antihypertensive medications need only demonstrate that they are effective in lowering blood pressure and do not have to show a reduced incidence of major vascular events. While early endpoints are widely used in many diseases to screen for activity – in cancer – the obvious such endpoint is tumour response – their use in Phase III studies to make definitive determinations about therapeutic efficacy is more controversial.

The example of blood pressure effectively illustrates the primary rationale for using surrogate endpoints. They provide potential for accelerating evaluation of new therapies, since use of earlier endpoints implies more rapid completion of trials. But there are other reasons why surrogate endpoints might be desirable. They might be observable more easily or less invasively or less expensively than the 'true' outcome. Studies of nutritional interventions to avert loss of body mass, for example, have often relied on such surrogate measures as weight and anthropometric measures such as skinfold thickness, since the 'gold standard' measures of body mass are exceedingly complex and the availability of the necessary equipment for such testing is quite limited. Yet another reason for the use of surrogate endpoints, one that is particularly relevant to cancer studies, is the 'competing risks' problem when a long-term endpoint such as survival is used. If a substantial proportion of the study population might be expected to die of causes unrelated to the disease under study, an earlier endpoint might actually provide a more precise estimate of the treatment effect. Even when most patients will eventually succumb to the disease in question, survival comparisons could become clouded by effects of concomitant or salvage therapies administered later in the course of treatment.

Because cancer is a life-threatening disease, the endpoint of primary interest in studies to evaluate and/or compare therapeutic strategies must be mortality. For many types of cancer, and for most cancers diagnosed at advanced stages of disease, it is unfortunately the case that survival is a perfectly feasible study endpoint because of the short life expectancy following diagnosis of these neoplasms and the minimal efficacies of available treatments. In other situations, however, survival studies will take many years to complete, and will require very large sample sizes to offset the expected dilution of observed differences due to, for example, use of salvage therapies or competing risks of death from other causes. This is frequently the case in studies of adjuvant therapies, particularly when the initial 'curative' therapy, usually surgery or radiotherapy, is substantially efficacious. Assessing survival may also be problematic in diseases such as head and neck cancer, in which patients are at risk of death from non-cancer causes.

There are two major problems with the use of surrogate endpoints to make definitive decisions about drugs. The first is that a drug may have the desired effect on the surrogate, but may have a limited effect on the clinical outcome of interest, or may have other adverse effects that outweigh the benefit, so that the ultimate clinical impact is null or even negative. An example going back some years is diethylstibestrol (DES) for treatment of prostate cancer. A review of several trials performed by the Veterans Administration between 1960 and 1975 showed that high doses of DES clearly induced greater tumour shrinkage and delayed progression of disease compared to a low dose of DES and a placebo. Nevertheless, overall survival was definitely better on the latter arms, due almost surely to the cardiotoxicity of this agent (Byar, 1973). Another even more dramatic example in a non-cancer context was the finding in a double-blind randomised trial by The Cardiac Arrhythmia Suppression Trial (CAST) Investigators (1989) that drugs that were effective in preventing arrhythmias in patients recovering from a myocardial infarction were associated with higher mortality, despite the fact that arrhythmias are known to be associated with poorer survival.

The converse may occur; all or part of a drug's benefit may not be mediated through the proposed surrogate so that the treatment effect may be underestimated if the true outcome is not assessed. In a recent study, interferon $\gamma$ for chronic granulomatous disease was found to be highly efficacious in protecting against the occurrence of serious bacterial infections, but the laboratory markers that had been thought to mediate the utility of this therapy were unaffected (The International Chronic Granulomatous Disease Cooperative Study Group, 1991).

The second problem is that a surrogate that appears appropriate to evaluate one class of treatments may not be appropriate to evaluate other treatments with different mechanisms of action. If treatments are interrupting disease via different mechanisms, there is no guarantee that a more proximal endpoint will equally well reflect the effects of these treatments. This problem may not be a serious one for tumour response, the prototype surrogate endpoint for cancer studies, since whatever the mechanism of action, tumour growth must be thwarted in order to improve ultimate outcome. It may, however, be of more concern for laboratory markers or other less direct measures of neoplastic activity, and may be particularly worrisome when we are comparing biologic or hormonal agents with more traditional cytotoxic therapy. A related issue is knowing exactly how to use such a marker to assess benefit. Is it the size of the change (absolute or relative to baseline level) that is important, or whether normal levels are achieved (and/or for how long), or the proportion of patients achieving a desired level? The size of the treatment benefit, and therefore the interpretation of the results of comparative trials, may be very dependent on the way the endpoint is defined.

Received and accepted 4 June 1993.

As the examples demonstrate, these problems are by no means purely theoretical. Fatal adverse effects are sometimes observed in studies evaluating adjuvant therapies for surgically resected tumours, precisely the type of long-term study in which intermediate endpoints would be attractive. In these studies, tumour recurrence is often the primary endpoint. This is a sensible approach, since the intent of adjuvant therapy is to improve the probability that the patient has in fact been cured of neoplastic disease. Nevertheless, the importance of obtaining the ultimate survival results cannot be overstated. The serious side effects, including drug-induced leukaemias, that have been reported for some of these agents (Reimer *et al.*, 1977; Bergsagel *et al.*, 1979; Coltman & Dixon, 1982; Boice *et al.*, 1983) point to the need to verify overall benefit with respect to survival.

An important practical issue concerning the use of surrogate endpoints is that of measurement variability. Other than death, all outcomes are assessed with some degree of uncertainty, due to some combination of measurement error and inherent within-subject biologic variability. The level of uncertainty of any given measure must be taken into account when considering its potential reliability as a trial endpoint. The imprecision of manual and radiologic measurements of tumour size has been well documented (Moertel & Hanley, 1976; Lavin & Flowerdew, 1980; Warr *et al.*, 1984); laboratory measures are likely to be at least as variable. While variability can always be reduced by replicate measurements at the defined time points, this approach is often impractical because of financial constraints as well as patient unwillingness to provide multiple samples. The variability of an outcome measure should be an important factor in any decision to use this measure as a basis for definitive evaluation of new therapies, especially when studies are non blinded and when the determination of the measure is at all subjective.

Recently, the use of laboratory markers as trial endpoints has received a great deal of discussion. Some widely used markers such as carcinoembryonic antigen (CEA) in colorectal cancer and CA 125 in ovarian cancer have been shown to be inadequately predictive for this purpose (Fayers *et al.* (1993), Ellenberg & Hamilton (1989), NIH Consensus Conference (1981)). There has been increasing interest in prostate-specific antigen (PSA) which has been shown to be highly correlated with tumour volume in men with prostatic cancer (Stamey *et al.*, 1987). Since much prostate cancer is non measurable, the availability of a reliable laboratory marker would greatly expand the patient population for Phase II studies to determine tumour activity. For definitive evaluation of clinical benefit, however, it is less clear that this marker or other markers will obviate the need to perform survival studies. While there are ample data to demonstrate the prognostic value of PSA, as exist for CEA and CA 125 as well, data demonstrating that advanced-stage patients who experience the largest treatment-induced reductions in PSA level, or those early patients who experience treatment-prolonged intervals of PSA negativity, will in fact have a lower risk of death, are only now beginning to emerge (Gerber *et al.*, 1990; Seidman *et al.*, 1992; Scher *et al.*, 1992). Until such data are fully available and are shown to be consistent across treatments, determining relative efficacy of therapeutic approaches on the basis of PSA will remain problematic.

These issues have been widely discussed in the AIDS context (Weiss & Mazade, 1990; Machado *et al.*, 1990; Jacobson *et al.*, 1991; Ellenberg, 1991; Lagakos & Hoth, 1992). The CD4+ lymphocyte count is a well documented predictor of outcome in HIV-infected patients, but changes in this marker do not appear to be as highly correlated with clinical progression and survival as one would like in order to use this marker as a basis for evaluating and comparing therapeutic efficacy (De Gruttola *et al.*, in press; Tsiatis *et al.*, 1992; Choi *et al.*, 1993). This may be partially due to the fact that measurements of CD4+ cells are known to be highly variable (Weiss & Mazade, 1990; Taylor *et al.*, 1989; Gelman *et al.*, in press). However, the recently reported result that in a large placebo-controlled trial of zidovudine in asymptomatic HIV-infected individuals, the CD4+ count was affected, but clinical progression and mortality were not (Aboulker & Swart, 1993), raises more doubts that this marker can serve as a reliable surrogate for clinical outcomes.

Nevertheless, as in prostate cancer, changes in the marker are well established as indicators of advancing disease, and are frequently used by physicians to determine when modification of the therapeutic approach may be required. In this situation it becomes more difficult to conduct trials using clinically meaningful endpoints, since changes in therapy mandated by these earlier marker events may blur any comparison based on later clinical endpoints.

In both AIDS and cancer, there exists a real sense of urgency to evaluate promising therapies and to make widely available those that prove efficacious. There are certainly risks inherent in waiting longer for answers from survival-based trials; current patients may be deprived of improved treatments. Such concerns must be weighed against the risk of adopting therapies on the basis of early data without the assurance that current as well as future patients will ultimately benefit from them.

### References

ABOULKER, J.-P., SWART, A.M., ON BEHALF OF THE CONCORDE COORDINATING COMMITTEE (1993). Preliminary analysis of the Concorde trials. (Letter to the Editor). *Lancet*, **341**, 889-890.

BERGSAGEL, D.E., BAILEY, A.J., LANGLEY, G.R. MACDONALD, R.N., WHITE, D.F. & MILLER, A.B. (1979). The chemotherapy of plasma-cell myeloma and the incidence of acute leukemia. *N. Eng. J. Med.*, **301**, 743-748.

BOICE, J.D., GREENE, M.H., KILLEN, J.Y., ELLENBERG, S.S., KEEHN, R.J., MCFADDEN, E., CHEN, T.T. & FRAUMENI, J.F. (1983). Leukemia and preleukemia after adjuvant treatment of gastrointestinal cancer with semustine (Methyl-CCNU). *N. Engl. J. Med.*, **309**, 1079-1084.

BYAR, D.P. (1973). The Veterans Administration Cooperative Urological Research Group's studies of cancer of the prostate. *Cancer*, **32**, 1126-1130.

CHOI, S., LAGAKOS, S.W., SCHOOLEY, R.T. & VOLBERDING, P.A. (1993). CD4+ lymphocytes are an incomplete surrogate marker of clinical progression in persons with asymptomatic HIV infection taking Zidovudine. *Ann. Int. Med.*, **118**, 674-680.

COLLINS, R.C., PETO, R., MACMAHON, S., HEBERT, P., FIEBACH, N.H., EBERLEIN, K.A., GODWIN, J., QIZILBASH, N., TAYLOR, J.O. & HENNEKENS, C.H. (1990). Blood pressure, stroke and coronary heart disease. *Lancet*, **335**, 827-838.

COLTMAN, C.A. Jr & DIXON, D.O. (1982). Second malignancies complicating Hodgkin's disease: a Southwest Oncology Group 10-year followup. *Cancer Treat. Rep.*, **66**, 1023-1033.

DAHLOF, B.J., LINDHOLM, L.H., HANSSON, L., SCHERSTEN, B., EKBOM, T. & WESTER, P.O. (1991). Morbidity and mortality in the Swedish Trial in Old Patients with Hypertension (STOP-Hypertension). *Lancet*, **338**, 1281-1285.

DE GRUTTOLA, V., WULFSOHN, M., FISCHL, M. & TSIATIS, A. (1993). Modeling the relationship between survival and CD4-CD4-lymphocytes in patients with AIDs and AIDS-Related Compex. *J. Acquir. Immune Defic. Syndr*, (in press).

ELLENBERG, S.S. & HAMILTON, J.M. (1989). Surrogate endpoints in clinical trials: cancer. *Stat. in Med.*, **8**, 405-413.

ELLENBERG, S.S. (1991). Surrogate end points in clinical trials. *BMJ*, **302**, 63-64.

FAYERS, P.M., RUSTIN, G., WOOD, R., NELSTROP, A., LEONARD, R.C.F., WILKINSON, P., CRUICKSHANK, D., MCALLISTER, E.J., REDMAN, C.W.E., PARKER, D., SCOTT, I.V., SLEVIN, M.L., ROULSTON, J.E., ATKINSON, J., ON BEHALF OF THE MRC WORKING PARTY ON GYNAECOLOGICAL CANCER (1993). The prognostic value of serum CA125 in patients with advanced ovarian carcinoma: an analysis of 573 patients. *Int. J. Gyn. Canc.* (in press).

GELMAN, R., CHENG, S.C., KIDD, P., WAXDAL, M. & KAGAN, J. (1993). Assessment of the effects of instrumentation, monoclonal antibody, and fluorochrome on flow cytometric immunophenotyping: a report based on two years of the NIAID DAIDS flow cytometry quality assessment program. *Clin. Immunol. Immunopathol.* (in press).

GERBER, G.S. & CHODAK, G.W. (1990). Prostate specific antigen for assessing response to ketoconazole and prednisone in patients with hormone refractory metastatic prostate cancer. *J. Urol.*, **144**, 1177.

JACOBSON, M.A., BACCHETTI, P., KOLOKATHIS, A., CHAISSON, R.E., SZABO, S., POLSKY, B., VALAINIS, G.T., MILDVAN, D., ABRAMS, D., WILBER, J., WINGER, E., SACKS, H.S., HENDRICKSON, C. & MOSS, A. (1991). Surrogate markers for survival in patients with AIDS and AIDS related complex treated with zidovudine. *BMJ*, **302**, 73–78.

LAGAKOS, S.W. & HOTH, D.F. (1992). Surrogate markers in AIDS: Where are we? Where are we going? *Ann. Int. Med.*, **116**, 599–601.

LAVIN, P.T. & FLOWERDEW, G. (1980). Studies in variation associated with the measurement of solid tumours. *Cancer*, **46**, 1286–1290.

MACHADO, S.G., GAIL, M.H. & ELLENBERG, S.S. (1990). On the use of laboratory markers as surrogates for clinical endpoints in the evaluation of treatments for HIV infection. *J. Acquir. Immune Defic. Syndr.*, **3**, 1065–1073.

MOERTEL, C.G. & HANLEY, J.A. (1976). The effect of measuring error on the results of therapeutic trials in advanced cancer. *Cancer*, **38**, 388–394.

REIMER, R.R., HOOVER, R., FRAUMENI, J.F. Jr & YOUNG, R.C. (1977). Acute leukemia after alkylating-agent therapy of ovarian cancer. *N. Eng. J. Med.*, **297**, 177–181.

SCHER, H.I., CURLEY, T., YEH, S., IVERSON, J.M., O'DELL, M. & LARSON, S.M. (1992). Therapeutic alternatives for hormone-refractory prostatic cancer. *Sem. Urol.*, **10**, 55–64.

SEIDMAN, A.D., SCHER, H.I., PETRYLAK, D., DERSHAW, D.D. & CURLFY, T. (1992). Estramustine and vinblastine: use of prostatic specific antigen as a clinical trial endpoint for hormone refractory prostatic cancer. *J. Urol.*, **147**, 931–934.

SHEP COOPERATIVE RESEARCH GROUP (1991). Prevention of stroke by antihypertensive drug treatment in older persons with isolated systolic hypertension. *JAMA*, **265**, 3255–3264.

STAMEY, T.A., YANG, N., HAY, A.R., MCNEAL, J.E., FREIHA, F.S. & REDWINE, E. (1987). Prostate-specific antigen as a serum marker for adenocarcinoma of the prostate. *N. Eng. J. Med.*, **317**, 909–916.

TAYLOR, J.M.G., FAHEY, J.L., DETELS, R. & GIORGI, J.V. (1989). CD4 percentage, CD4 number, and CD4:CD8 ratio in HIV infection: which to choose and how to use. *J. Acquir. Immune Defic. Syndr.*, **2**, 114–124.

THE CARDIAC ARRHYTHMIA SUPPRESSION TRIAL (CAST) INVESTIGATORS (1989). Preliminary report: effect of encainide and flecainide on mortality in a randomized trial of arrhythmia suppression after myocardial infarction. *N. Eng. J. Med.*, **321**, 406–412.

THE INTERNATIONAL CHRONIC GRANULOMATOUS DISEASE COOPERATIVE STUDY GROUP (1991). A controlled trial of interferon $\gamma$ to prevent infection in chronic granulomatous disease. *N. Eng. J. Med.*, **324**, 509–516.

TSIATIS, A., DAFNI, U., DE GRUTTOLA, V., PROPERT, K., STRAWDERMAN, R. & WULFSOHN, M. (1992). The relationship of CD4 counts over time to survival in patients with AIDS: Is CD4 a good surrogate marker? In AIDS Epidemiology: Methodological Issues, Jewell, N.P., Dietz, K. & Farewell, V.T. (eds.), Birkhauser: Boston.

WARR, D., MCKINNEY, S. & TANNOCK, I. (1984). Influence of measurement error on assessment of response to anticancer chemotherapy: proposal for new criteria of tumour response. *J. Clin. Onc.*, **2**, 1040–1046.

WEISS, R. & MAZADE, L. (eds.) (1990). Surrogate Endpoints in Evaluating the Effectiveness of Drugs Against HIV Infection and AIDs. National Academy Press: Washington, D.C. (Institute of Medicine conference summary).

SUMMARY OF AN NIH CONSENSUS STATEMENT (1981). Carcinoembryonic antigen: its role as a marker in the management of cancer. *BMJ*, **282**, 373–375.